# Supplementary material for: The interactome of intact mitochondria by cross-linking mass spectrometry provides evidence for coexisting respiratory supercomplexes
Source: Mol Cell Proteomics. 2017 Dec 8;17(2):216–32. doi: 10.1074/mcp.RA117.000470 (PMC5795388; doi:10.1074/mcp.RA117.000470)
Supplement: Supplemental Data [file supp_RA117.000470_133922_0_supp_23978_fzffwf.zip › spectra_annotation/mito_spectra_annotation/38-1-2-1-6-1.pdf]

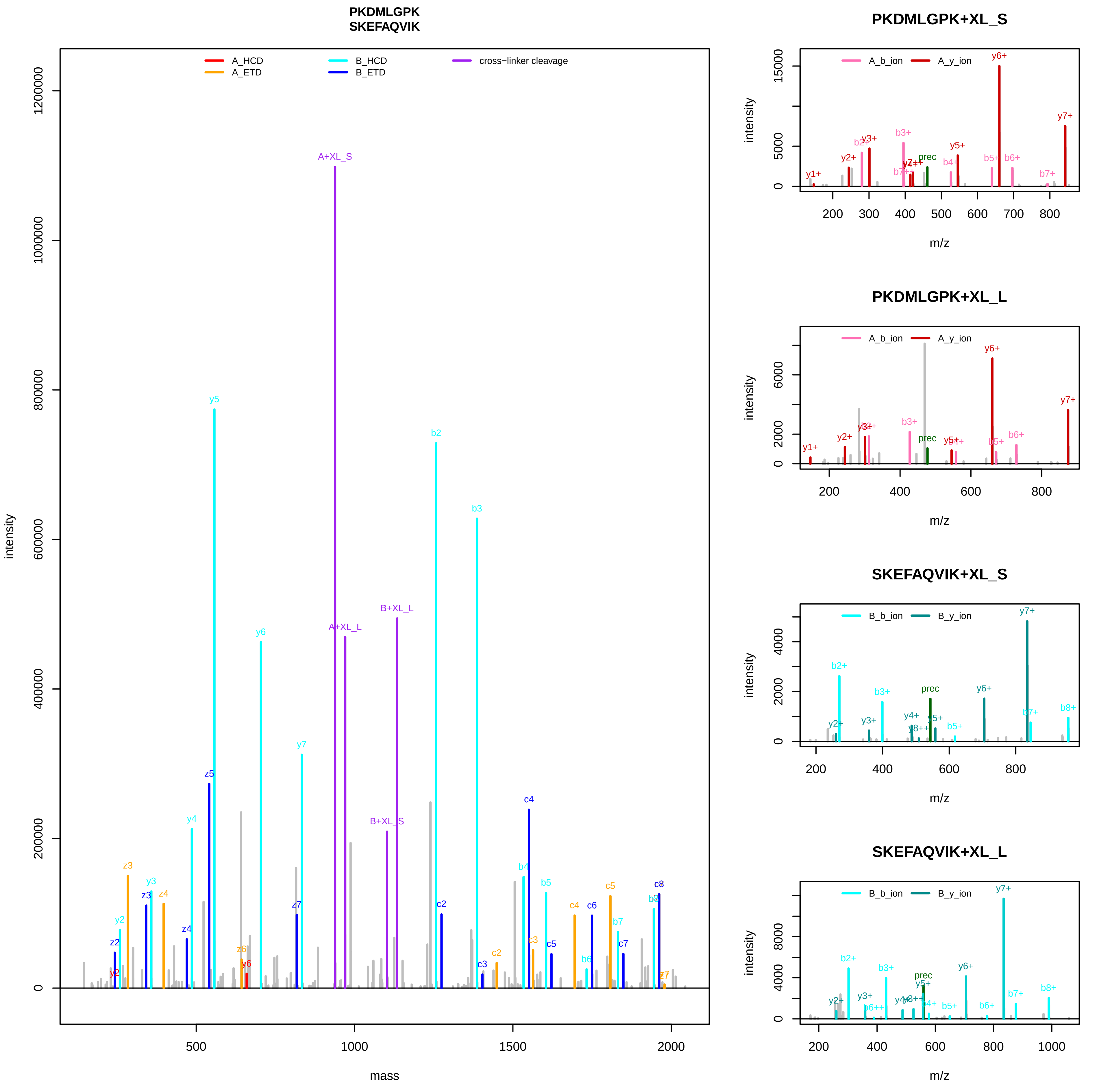
PKDMLGPK
SKEFAQVIK
A_HCD
A_ETD
B_HCD
B_ETD
cross−linker cleavage
intensity
mass
A+XL_S
A+XL_L
B+XL_L
B+XL_S
y5
y6
y7
y4
y3
y2
z2
z3
z4
z5
z7
z3
z4
z6
y6
y2
b2
b3
b4
b5
b6
b7
c2
c3
c4
c5
c7
c8
c2
c3
c4
c5
z7
PKDMLGPK+XL_S
PKDMLGPK+XL_L
SKEFAQVIK+XL_S
SKEFAQVIK+XL_L
A_b_ion
A_y_ion
B_b_ion
B_y_ion
prec
m/z
